# Denosumab-Induced Rebound Hypercalcemia Treated With Bisphosphonates in a Pediatric Patient

**DOI:** 10.1210/jcemcr/luad133

**Published:** 2023-10-28

**Authors:** Anne Gandolfi, Sarah Shaaban

**Affiliations:** Department of Pediatrics, Rush University Medical Center, Rush Pediatric Residency Program, Chicago, IL 60612, USA; Department of Pediatric Endocrinology, Rush University Medical Center, Chicago, IL 60612, USA

**Keywords:** denosumab, rebound hypercalcemia, aneurysmal bone cyst, zoledronic acid

## Abstract

Denosumab is a RANK-L inhibitor used off-label as a treatment for a variety of pediatric bone disorders, including aneurysmal bone cysts (ABC). Rebound hypercalcemia is a known side effect after denosumab therapy and is more commonly reported in pediatric patients. Although there are no established treatment guidelines, denosumab-induced rebound hypercalcemia is usually managed with a combination of intravenous fluids, diuretics, corticosteroids, denosumab, and/or bisphosphonates. We present the case of a 10-year-old female patient with history of a right sacral ABC treated with denosumab who presented with recurrent episodes of rebound hypercalcemia beginning 3 months after denosumab cessation. After the third hospitalization for hypercalcemia, which was treated with zoledronic acid, normocalcemia was achieved. This case demonstrates an increasingly recognized side effect of denosumab therapy that occurs mainly in skeletally immature patients and presents a possible approach to initial therapy of rebound hypercalcemia with a long-acting bisphosphonate.

## Introduction

Rebound hypercalcemia following treatment with denosumab is a known side effect that is more frequently reported in skeletally immature patients [1-3]. Currently, no pediatric guidelines exist for the treatment of denosumab-induced rebound hypercalcemia. In this case, we present a 10-year-old female patient with history of aneurysmal bone cyst (ABC) initially treated with surgical intervention, followed by a 1-year course of denosumab, who developed recurrent rebound hypercalcemia requiring 3 hospitalizations starting 3 months after the last dose of denosumab. A combination of intravenous fluids, diuretics, corticosteroids, and bisphosphonates was used for treatment. The hypercalcemia resolved after treatment with zoledronic acid during her last hospitalization.

## Case Presentation

A 10-year-old female individual with no prior medical history initially presented at age 8 with progressive low back pain, right foot drop, and refusal to bear weight on the right lower extremity. Magnetic resonance imaging showed a multiloculated cystic lesion within the right sacrum with encroachment into the S1 neural foramina. She underwent urgent vascular embolization of the medial sacral artery, curettage, and S1 decompression, with intraoperative pathology confirming the diagnosis of an ABC. She re-developed symptoms several months later and required ABC aspiration with bone cement placement. The multidisciplinary tumor board recommended medical treatment with denosumab due to failure of surgical management and ongoing symptoms. Prior to denosumab initiation, laboratory tests were notable for hypophosphatemia (3.1 mg/dL; reference range, 4.5-6.5 mg/dL; 1.00 mmol/L), hyperchloremia (114 mmol/L; reference range, 99-108 mmol/L; 114 mEq/L), and low 25-hydroxy vitamin D (23 ng/mL; reference range, 30-80 ng/mL; 57.41 nmol/L). Her complete blood count, magnesium, and the remainder of the complete metabolic panel were unremarkable.

Denosumab therapy was initiated, with 60 mg per dose for 22 total doses based on a previously described protocol: weekly for 4 doses, every 4 weeks for 12 doses, and every 8 weeks for 6 doses [4]. Calcium carbonate 24 mg/kg/day of elemental calcium and cholecalciferol 1000 IU daily were initiated prophylactically at the start of denosumab therapy. After 3 months of therapy, she was reporting no pain, was able to bear weight on her right lower extremity, and she had improved range of motion noted at physical therapy sessions. After the last denosumab dose, calcium carbonate was continued for one additional month and cholecalciferol was continued due to 25-hydroxy vitamin D level of 15.7 ng/mL (reference range, 30-100 ng/ mL; 39.19 nmol/L). Serum calcium levels remained normal, ranging from 8.4 to 10.1 mg/dL (2.10 to 2.52 mmol/L; reference range, 8.0-11.0 mg/dL) throughout denosumab therapy. Monthly labs were collected starting 2 months after the last denosumab dose to monitor for rebound hypercalcemia.

## Diagnostic Assessment

Monitoring labs collected 108 days after the last denosumab dose showed hypercalcemia (11.6 mg/dL, reference range, 8.0-11.0 mg/dL; 2.9 mmol/L), hyperphosphatemia (6.2 mg/dL; reference range, 3.7-5.6 mg/dL; 2.0 mmol/L), and low 25-hydroxy vitamin D (20.9 ng/mL; 52.17 nmol/L). That same day, she developed progressive nausea, vomiting, abdominal pain, diarrhea, and fatigue. She presented to the emergency room on day 3 of symptoms.

Her initial exam was notable for elevated blood pressure (122/75 mmHg), tachycardia (heart rate 110 bpm), and dry mucous membranes. Blood tests demonstrated serum calcium of 15.3 mg/dL (reference range, 9.2-10.6 mg/dL; 3.83 mmol/L), albumin 4.3 g/dL (reference range, 3.8-4.5 g/dL; 43 mg/L), ionized calcium 1.65 mmol/L (reference range, 1.12-1.23 mmol/L; 6.60 mg/dL), parathyroid hormone related protein of <2.0 pmol/L (reference range, <2.0 pmol/L), and appropriately suppressed intact parathyroid hormone (4.6 pg/mL; reference range, 8.0-85.0 pg/mL). Creatinine was within reference range (0.8 mg/dL; reference range, 0.5-0.8 mg/dL; 70.72 umol/L) but elevated from her baseline ranging from 0.3-0.59 mg/dL. Urine calcium was appropriately detectable at 16.3 mg/dL with a urine calcium to urine creatinine ratio of 1.63 mg/mg (reference range, <0.22 mg/mg). Thyroid function tests and morning cortisol were within normal limits.

## Treatment

Initial management included intravenous 0.9% saline with 5% dextrose at a rate of 3.55 to 4.75 mL/kg/h and furosemide. Furosemide was held intermittently to prevent exacerbation of acute kidney injury, with creatinine peaking at 1.16 mg/dL (reference range, 0.52-0.69 mg/dL; 102.54 umol/L) on hospital day 2. A renal ultrasound was obtained due to concern for nephrocalcinosis, which was unremarkable. Due to persistence of hypercalcemia, pamidronate was initiated on hospital day 1. The recommended pamidronate dose for severe hypercalcemia is 1.5-2 mg/kg. In collaboration with our pediatric pharmacist, we decided on an initial dose of 1 mg/kg due to her relative kidney injury.

## Outcome and Follow-Up

Ionized calcium gradually normalized by hospital day 4. Two episodes of hypocalcemia occurred during hospitalization, both of which normalized without intervention. She was discharged on hospital day 7 with cholecalciferol 1000 IU daily.

**Figure 1.**
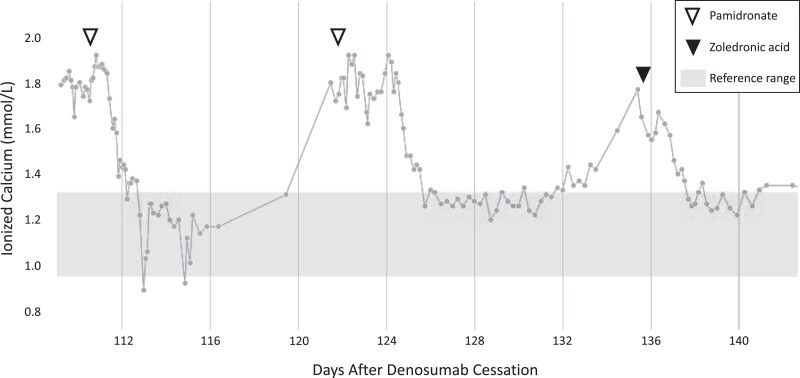
Serum ionized calcium levels and bisphosphonate treatments during hospital admissions.

Five days after discharge (121 days after denosumab cessation), she was readmitted for hypercalcemia (ionized calcium 1.80 mmol/L; reference range, 1.12-1.23 mmol/L; 7.20 mg/dL) associated with abdominal pain and emesis. She was treated with intravenous lactated ringers with 5% dextrose at a rate of 4.80 mL/kg/h, furosemide, a second pamidronate 1 mg/kg infusion, and methylprednisolone 1 mg/kg twice daily for 5 days. Two days after discharge from the second hospitalization (135 days after denosumab cessation), she was admitted for asymptomatic hypercalcemia detected on outpatient laboratory monitoring: ionized calcium 1.77 mmol/L (reference range, 0.95-1.32 mmol/L; 7.08 mg/dL). She was treated with one dose of zoledronic acid 0.03 mg/kg on hospital day 1, methylprednisolone 1 mg/kg twice daily for 3 days (discontinued early due to hypertension), and intravenous 0.9% saline and 0.45% saline with 20 mEq potassium chloride at a rate of 3.61 to 4.77 mL/kg/h. Calcium levels normalized by hospital day 2 and she was discharged on hospital day 6. Ionized calcium trends during the 3 hospitalizations are demonstrated graphically in Fig. 1. She has remained eucalcemic on outpatient laboratory monitoring as of 6 months post hospital discharge at the time of this report.

## Discussion

Denosumab is a human monoclonal antibody that binds and sequesters RANK-L, thereby preventing the RANK-L/RANK interaction that leads to osteoclast differentiation and activity. Unlike bisphosphonates which bind to hydroxyapatite in the bone matrix for many years, denosumab does not bind to the bone matrix and therefore its effects are reversible. Rebound hypercalcemia is a known adverse effect of denosumab therapy. Although the exact mechanism is not clear, it is hypothesized to be caused by rapid bone resorption following recovery of osteoclast activity. Increased proportions of osteoclast precursors have been detected in peripheral blood mononuclear cells following treatment with denosumab [5]. Denosumab results in the formation of sclerotic metaphyseal bands that fade after treatment, and it is thought that mineral release from these bands contributes to rebound hypercalcemia [2].

The pharmacokinetics and pharmacodynamics of denosumab have been studied in adults but not children [2]. Rebound hypercalcemia following denosumab therapy has been more frequently reported in pediatric patients. In a systematic review of 49 cases of rebound hypercalcemia following denosumab therapy, 40 of those patients (82%) were skeletally immature [1]. In addition, the time interval from denosumab discontinuation to hypercalcemia was shorter in skeletally immature patients (interquartile range, 3-5 months), and their initial calcium levels were higher [1]. In a review of 43 patients with ABCs treated with denosumab, 6 developed rebound hypercalcemia, and they were all pediatric patients [3]. It is suspected that the rebound effect occurs more frequently in children than adults due to higher rates of bone metabolism and growth in children. Horiuchi et al estimated that rebound hypercalcemia occurs in 45% of skeletally immature patients treated with denosumab [1].

ABCs are benign bone lesions that occur most frequently in the first 2 decades of life. Although benign, they often cause a significant amount of local destruction and clinical symptoms, including pain and neurologic effects. Treatment options for ABCs include surgical resection, intralesional curettage, sclerotherapy, radiotherapy, and selective arterial embolization [6]. Axial ABCs are particularly challenging to treat with these modalities due to difficulty in accessing the tumor and increased risk of damaging neighboring structures [7]. Bone resorption in ABCs is mediated by RANK/RANK-L interaction between osteoclast-like multinucleated giant cells and neoplastic stromal cells [6]. Therefore, denosumab has been used to prevent this interaction, particularly in ABCs not amenable to traditional therapies. In a review of 43 patients with ABCs treated with denosumab, all experienced clinical improvement, and the vast majority had radiologic improvement, when reported [3]. Similar results of clinical and radiographic improvement were reported in a multicenter retrospective study of 5 pediatric patients with ABCs treated with denosumab [7] and a review of 30 patients with ABCs (20 of whom were younger than 18 years) treated with denosumab [6].

Although no established treatment recommendations exist, the management of denosumab-induced rebound hypercalcemia typically involves a combination of intravenous hydration, diuretics, calcitonin, corticosteroids, bisphosphonates, and/or repeat dosing of denosumab. We elected to use a combination of hydration, furosemide, corticosteroids, pamidronate, and zoledronic acid. Calcitonin was considered but not pursued due to risk of tachyphylaxis and unavailability in the hospital formulary at the time of this patient's admission. Similarly, denosumab was avoided due to concern for inciting further episodes of rebound hypercalcemia. Because steroids inhibit enteral absorption of calcium, we administered a course of steroids after her second admission to mitigate further exacerbation of hypercalcemia. The patient's first 2 episodes were managed with pamidronate, a bisphosphonate with a half-life of approximately 28 hours. Hypercalcemia recurred 11 days (∼9 half-lives) and 14 days (∼12 half-lives) after pamidronate infusions, respectively. Her third episode was treated with zoledronic acid, a bisphosphonate with a half-life of 146 hours. Because long-lasting normocalcemia was achieved after administration of zoledronic acid, we hypothesize that initial management with a bisphosphonate with a longer half-life may have been more effective in preventing subsequent episodes of hypercalcemia. Alternatively, the rebound resorptive effect post denosumab may have diminished by the end of her third hospitalization, regardless of the type of bisphosphonate that was used. Another interesting approach is treatment targeting prevention of anticipated rebound hypercalcemia. Seale et al reported a strategy involving alternating doses of denosumab and zoledronic acid every 3 months in a young boy with osteogenesis imperfecta type IV after an episode of denosumab-induced rebound hypercalcemia [8]. The boy had no further episodes of hypercalcemia. Harcus et al administered ibandronate at the time of denosumab cessation in a 13-year-old patient with a giant cell tumor, but the patient still developed hypercalcemia 3 months later [9]. Outside of a few case reports, there are no comprehensive literature reports or guidelines regarding the prevention of rebound hypercalcemia.

There are currently no guidelines regarding the timing or frequency of laboratory monitoring during or following denosumab therapy. Hypercalcemia has been reported to occur from less than 1 month to 7 months following denosumab cessation [1]. It is difficult to recommend a frequency of monitoring since hypercalcemia can be rapidly progressive within days after a normal calcium level. For this reason, patients and families should be educated on the signs and symptoms of hypercalcemia to facilitate timely recognition and treatment. One case reported a progressive increase in urinary calcium to creatinine ratio, parathyroid hormone suppression, and a decline in bone turnover markers prior to the development of hypercalcemia [8]. More research is needed on the potential of using these labs, rather than serum calcium alone, to predict impending hypercalcemia.

Because denosumab is a promising agent in the treatment of pediatric ABCs, it is important to better understand the risk factors, prevention, and best management for its adverse effect of rebound hypercalcemia. Early management with longer lasting bisphosphonates such as zoledronic acid may be beneficial in preventing repeat episodes of hypercalcemia. Future research should aim to further examine the role of zoledronic acid as a first-line treatment for rebound hypercalcemia after denosumab cessation.

## Learning Points

- Rebound hypercalcemia is a known side effect of denosumab therapy that is more frequently reported in skeletally immature patients and typically develops 3 to 5 months after denosumab discontinuation.
- Because denosumab-induced rebound hypercalcemia often presents with acute symptoms of hypercalcemia before it is detected on routine laboratory monitoring, it is important to educate patients and families on the symptoms of hypercalcemia so they can promptly seek treatment.
- Long-acting bisphosphonates, such as zoledronic acid, should be considered in the early management of denosumab-induced rebound hypercalcemia, as they may prevent subsequent episodes of rebound hypercalcemia.

## Data Availability

Data sharing is not applicable to this article as no datasets were generated or analyzed during the current study.

## Abbreviation

ABC: aneurysmal bone cyst
